# Cyclophosphazene Intrinsically Derived Heteroatom (S, N, P, O)-Doped Carbon Nanoplates for Ultrasensitive Monitoring of Dopamine from Chicken Samples

**DOI:** 10.3390/bios12121106

**Published:** 2022-12-01

**Authors:** Yasir Abbas, Naeem Akhtar, Sania Ghaffar, Ahlam I. Al-Sulami, Muhammad Asad, Muhammad Ehsan Mazhar, Farhan Zafar, Akhtar Hayat, Zhanpeng Wu

**Affiliations:** 1 State Key Laboratory of Organic-Inorganic Composites, Beijing University of Chemical Technology, Beijing 100029, China; 2 Institute of Chemical Sciences, Bahauddin Zakariya University (BZU), Multan 60800, Pakistan; 3 Department of Medicine and Surgery, Nishtar Medical University, Multan 60800, Pakistan; 4 Department of Chemistry, College of Science, University of Jeddah, Jeddah 21589, Saudi Arabia; 5 Department of Materials Science and Engineering, University of Science and Technology of China, Hefei 230026, China; 6 Department of Physics, Bahauddin Zakariya University (BZU), Multan 60800, Pakistan; 7 Department of Chemistry, Lahore Campus, COMSATS University Islamabad, Lahore 54000, Pakistan; 8 Interdisciplinary Research Centre in Biomedical Materials (IRCBM), COMSATS University Islamabad, Lahore 54000, Pakistan

**Keywords:** cyclophosphazene, intrinsic doping, carbon nanoplates, dopamine sensor

## Abstract

A novel, metal-free electrode based on heteroatom (S, N, P, O)-doped carbon nanoplates (SNPO-CPL) modifying lead pencil graphite (LPG) has been synthesized by carbonizing a unique heteroatom (S, N, P, O)-containing novel polymer, poly(cyclcotriphosphazene-co-2,5-dioxy-1,4-dithiane) (PCD), for precise screening of dopamine (DA). The designed electrode, SNPO-CPL-800, with optimized percentage of S, N, P, O doping through the sp2-carbon chain, and a large number of surface defects (thus leading to a maximum exposition number of catalytic active sites) led to fast molecular diffusion through the micro-porous structure and facilitated strong binding interaction with the targeted molecules in the interactive signaling transducer at the electrode–electrolyte interface. The designed SNPO-CPL-800 electrode exhibited a sensitive and selective response towards DA monitoring, with a limit of detection (LOD) of 0.01 nM. We also monitored DA levels in commercially available chicken samples using the SNPO-CPL-800 electrode even in the presence of interfering species, thus proving the effectiveness of the designed electrode for the precise monitoring of DA in real samples. This research shows there is a strong potential for opening new windows for ultrasensitive DA monitoring with metal-free electrodes.

## 1. Introduction

It has been reported that, every year nearly one million people commit suicide because of neurological disorders including Parkinson’s, schizophrenia, depression, restless leg syndrome, and bipolar disorder [1]. Significant efforts have been made to observe these disorders in the human body by monitoring various biomolecules, including DA, ascorbic acid (AA), uric acid (UA), glucose, and hydrogen peroxide. Accurate monitoring of these molecules not only assures proper treatment but also prevents various genetic diseases. DA is an important neurotransmitter, which regulates various functions of the central nervous system such as cognition, learning, mobility, and emotions in mammals [2]. Thus, developing a reliable system to monitor DA levels in real or physiological samples is of great interest.

Several analytical methods including capillary electrophoresis, Raman spectroscopy, high-performance liquid chromatography, fluorescence spectrometry, and the electrochemical technique have recently been widely applied to monitor DA levels. Among these, the electrochemical technique has attracted a great amount of attention because of its simplicity, precision, and, most importantly, commercial availability to the public [3]. Both, enzymatic and non-enzymatic electrochemical sensors have been widely applied to monitoring DA levels. Among these, enzyme-based electrochemical sensors have received much interest because of their tremendous selectivity and reliability in terms of reproducibility. However, issues regarding cost, stability, and their complex fabrication route have hampered the development of these enzyme-based sensors [3]. Thus, development of enzyme-free electrodes is of great need to increase stability, lower cost, and simplify fabrication.

A major challenge in developing electrochemical sensors for DA is to find a material that can selectively monitor DA even in the co-existence of electro-active species, including AA and UA [4]. To deal with this issue, two different strategies have been adopted: One is to add negatively charge species/polymers along with an electro-active catalyst at the surface of the electrode to repel negatively charged interferences. The other is to construct an electro-active catalyst that could easily separate the oxidation peak of co-existing species. Compared to the first, the second strategy has gained more interest because of the advantages of large surface area, high sensitivity, notable electrical features, excellent biocompatibility, and reliable reproducibility [5,6]. For example, several tailor-made nanoparticles and composites, including palladium nanoclusters [7], gold/silver nanoclusters [8], gold nanoslices [9], titanium dioxide-anchored graphene oxide nanosheets [10], NiO broccolis [11], Pt_3_Ni nanoalloy [11,12], platinum-nickel bimetallic/reduced graphene oxide [13], 3D porous graphene [14], and ZnO-polyaniline/reduced graphene oxide [12], have been successfully applied to monitor DA selectively.

Despite these developments, new designs for low-cost, straightforward, and user-friendly sensors are in high demand. Recently, heteroatom-doped, metal-free carbon-based materials have attracted considerable interest because of their high surface area with maximum fraction of catalytic active sites and increased surface defects [15]. Interestingly, it has been reported that the doping of heteroatoms, including sulfur (S), nitrogen (N), phosphorous (P), and boron, into carbon networks not only exerts a positive impact on the conductance and hydrophilicity but also increases the transportation capability of electrons [16]. In this regard, various polymers such as polyaniline, polypyrole, and polythiophene have been extensively reported as sources of N or S heteroatoms for the preparation of metal-free, heteroatom-doped carbon nanomaterials [17,18]. Similarly, polyaniline aerogel has been used as a source of N and P to fabricate three-dimensional N and P co-doped mesoporous nanocarbons with bifunctional catalytic activity [19]. Even though these polymer precursors can offer heteroatom-doped carbon, there is a need to develop polymer precursors that are easy to synthesize and offer a maximum number of heteroatoms with a large number of surface defects.

Herein, we report a facile template-free strategy to synthesize a metal-free S, N, P, and O doped carbon nanoplatelet (SNPO-CPL) electrocatalyst by carbonizing a novel compound, poly(cyclcotriphosphazene-co-2,5-dioxy-1,4-dithiane) (PCD) polymer. PCD has been fabricated using 1,4-dithiane-2,5-diol (DD) and hexachlorocyclotriphosphazene (HCCP) monomers. The PCD polymer was selected not only because of the availability of heteroatoms such as S, N, P, and O but also due to its high carbon content [20,21]. Direct carbonization of the as-produced PCD polymer at three different temperatures, including 600, 800, and 1000 °C, produced the SNPO-CPL catalyst and were named based on calcined temperature (SNPO-CPL-600, SNPO-CPL-800, and SNPO-CPL-1000). The as-obtained carbonized SNPO-CPL catalyst was further activated with 5 M KOH aqueous solution, dried at 80 °C overnight, and deposited at the surface of a lead pencil graphite (LPG) electrode. As-fabricated SNPO-CPL-800, with its plate-like morphology, fine-tuned tiny crystal particles, large carbon chain surface defects, optimized percentages of S, N, P, and O deponents, and more exposed active sites (along with the high electron transport capability of the LPG electrode), acts as an ideal tool due to its high selectivity and sensitivity for concurrent monitoring of DA, AA, and UA in the tertiary mixture. As far as we are aware, this research study is the first of its kind in relation to its innovative approach for real-time monitoring of DA from chicken samples through fabricated LPG electrodes modified with SNPO-CPL-800.

## 2. Experimental Section

### 2.1. Reagents and Materials

Tetrahydrofuran (THF), potassium hydroxide (KOH), xylene, and triethylamine (TEA) were obtained from Beijing Chemical Works, China. Prior to use, dry petroleum ether was used to recrystallize hexachlorocyclotriphosphazene (HCCP). 1,4-Dithiane-2,5-Diol (DD) was purchased from Alladin Chemicals in China. Local markets provided the chicken sample, while Sigma-Aldrich provided the uric acid (UA), L^+^-ascorbic acid (AA), phosphate buffer saline (PBS), and potassium ferricyanide (K_3_Fe(CN)_6_). All the chemicals were used without any additional purification.

### 2.2. Synthesis of Poly(cyclotriphosphazene)-1,4-dithiane-2,5-diol (PCD) Polymer

**Scheme 1:**
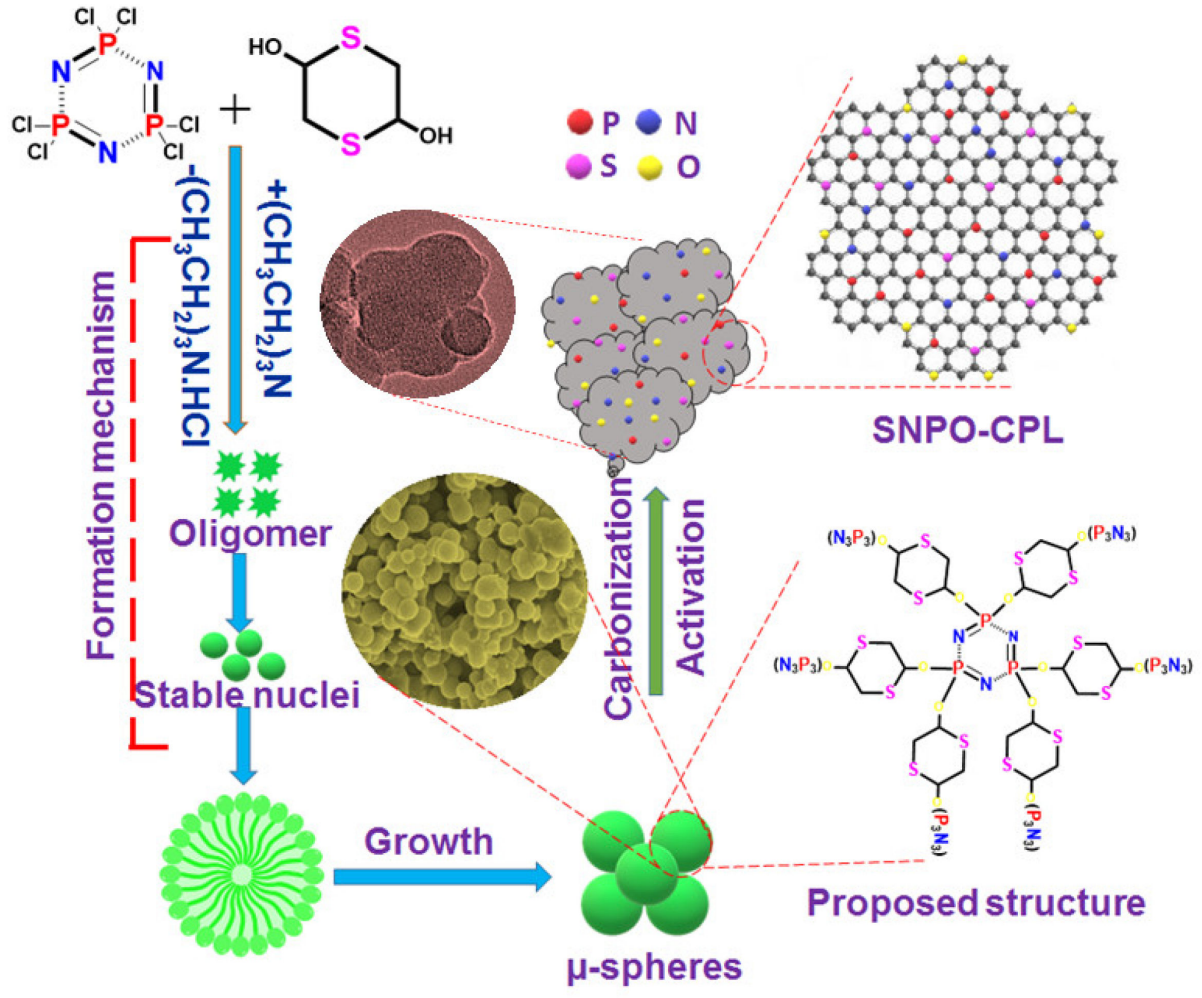
Schematic diagram showing fabrication route for the synthesis of SNPO-CPL. The as-obtained carbonized sample was further activated with 5 M KOH aqueous solution at 80 °C for 5 h with KOH/carbon ratio of 1. The activated product was first rinsed with deionized water before being dried at 80 °C overnight. Based on carbonization temperature, the obtained SNPO-CPL catalyst was named as SNPO-CPL-600. By adopting the same procedure, synthesized PCD were also carbonized at 800 and 1000 °C, separately, and the products were labeled as SNPO-CPL-800 and SNPO-CPL-1000, respectively.

The PCD polymer microspheres were synthesized analogous to the method previously reported by our group [22,23,24]. Briefly, a 100 mL mixture of xylene (25 mL) and THF (75 mL) was prepared. Next, 0.34 g of HCCP and 0.45 g of DD were dissolved in the prepared 100 mL mixture of xylene and THF in a conical flask (200 mL). Subsequently, TEA (5 mL) was added dropwise to the conical flask after ultrasonic treatment for 1 h. The resultant solution underwent ultrasonic treatment (150 W and 40 kHz) and was kept at ambient temperature overnight. The product was filtered to separate it, and it was then washed with ethanol and deionized water. The as-synthesized product was next subjected to drying in an oven at 80 °C. The schematic diagram for the complete fabrication process is shown in Scheme 1.

### 2.3. Synthesis of SNPO-CPL

SNPO-CPL catalyst was obtained through direct carbonization of as-synthesized PCD polymer under three temperatures, including 600, 800, and 1000 °C. Briefly, PCD (0.5 g) polymer was taken in a ceramic crucible and positioned in the furnace at 600 °C, having a ramp-up rate of 5 °C/min. Under the influence of N_2_ atmospheres, the carbonization was sustained for 6 h. Without lifting the lid of the furnace, the tube furnace spontaneously cooled to room temperature.

### 2.4. Electrochemical Measurements

All electrochemical experiments were performed with a Metrohm Autolab (Sr. # AUT50296, Herisau Switzerland) instrument, controlled by Nova 1.11 software, at room temperature. The electrochemical cell was a conventional three-electrode system, which consisted of a platinum wire as counter electrode, an Ag/AgCl as reference electrode, and a SNPO-CPL modified lead pencil graphite (LPG) electrode (LPG: 0.5 mm diameter and 40 mm length) as working electrode (Figure S1). For the modification of the working electrode, it was dipped in a 5 mL solution of SNPO-CPL (2 mg) and Milli-Q water for 4 h under room temperature. The modified electrode was further dried overnight and, before using it, the edge of the pencil lead electrode was cut down via a sharp blade to obtain the clean and smooth surface. The electrical contact of the electrode was made using Cu wire. DPV measurements were performed with a pulse height of 60, pulse distance of 100 ms, and at a constant scan rate of 100 mV/s.

### 2.5. Real Sample Analysis

Chicken sample was collected from the local market and prepared for DA analysis following the already reported protein precipitation approach with ZnCl_2_ [25]. Briefly, 5 g of chicken sample was cut and ground in 12 mL of PBS (0.1 M) into a fine paste with the help of mortar and pestle. The obtained paste was next mixed with ZnCl_2_ for occasional homogenizing. The pH of the mixture was adjusted to 7 with the help 0.1 M NaOH. The resulting mixture was then centrifuged for 20 min at 8000 rpm and filtered using a Millipore syringe filter. The resultant sample was next spiked with a known concentration of DA before applying for DA monitoring.

## 3. Results and Discussion

### 3.1. Structural and Morphological Assessment of PCD Polymer

**Figure 1:**
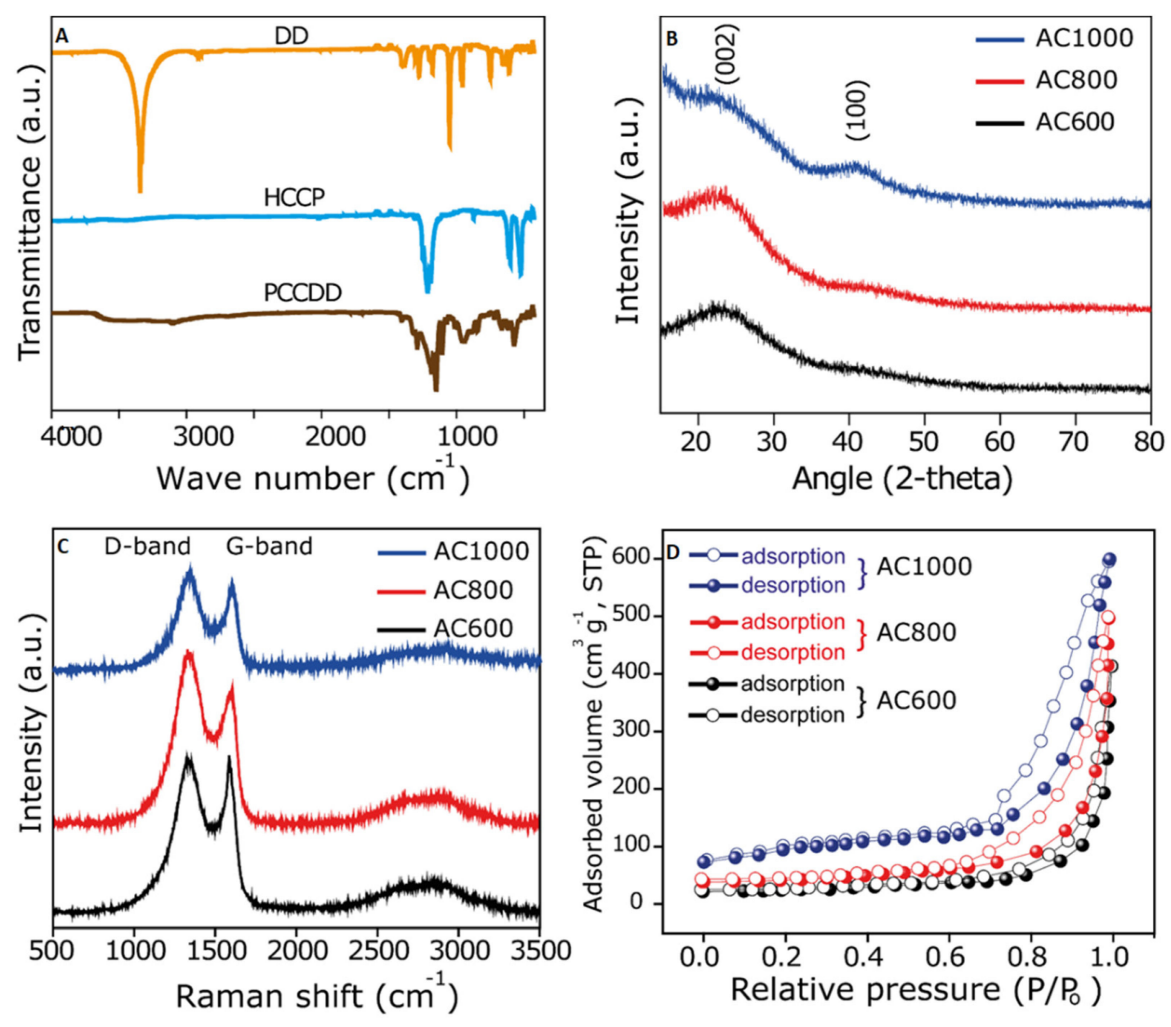
(**A**) FTIR analysis of DD monomer, HCCP monomer, and PCD polymer. (**B**) XRD, (**C**) Raman, and (**D**) N_2_ absorption-desorption isotherms of SNPO-CPL-600 (black line), SNPO-CPL-800 (red line), and SNPO-CPL-1000 (blue line), respectively.

FTIR characterizations were performed to evaluate the polymerization of HCCP and DD to form PCD polymer. Figure 1A shows the absorption bands of constituent monomers, DD, HCCP, and PCD polymer. The absorption bands at 780, 659, and 1420 cm^−1^ are attributed to C-H bending, C-S stretching of dithiane moiety, and aliphatic C-C stretching vibrational modes of DD, respectively. A strong absorption band at around 1728 cm^−1^ corresponds to the carbonyl stretching vibration of the ester group, while absorption bands at 2936 and 1130 cm^−1^ could be ascribed to aliphatic C-H stretching of the diacids/diols and C-O stretching. Additionally, a sharp absorption band at 3410 cm^−1^ corresponds to stretching vibrational modes of the OH^−^ group of DD, while peaks ranging between 580–1400 cm^−1^ are the characteristic absorption bands related to -C–C- and -S–C-. After polymerization, the distinctive signal at 3410 cm^−1^ totally vanishes, indicating that the OH^-^ groups have been removed [25]. The absorption bands ranging from 1100–1295 cm^−1^ correspond to vibrational modes of poly-phosphazene, while the absorption peak at 1250 cm^−1^ corresponds to the stretching vibration of the P=N of the cyclo-triphosphazene skeleton in the HCCP structure. A broad absorption band at 935 cm^−1^ shows bending vibrational mode, thus confirming the P-O-C formation. These distinctive forms unequivocally demonstrate the effective co-polymerization of the monomers. Similarly, XRD analysis (Figure S2) represents well-resolved diffraction peaks of (002) and (100) planes at representative 2θ value of 15 and 40, thus confirming the formation of PCD polymer. SEM images further revealed that the as-synthesized PCD polymer is composed of micro-spheres with size ranging from 0.2–0.5 µm (Figure S3).

### 3.2. Structural and Morphological Assessment of SNPO-CPL

XRD of the as-synthesized SNPO-CPL with carbonization temperature 600, 800, and 1000 °C depicts two diffraction peaks of (002) and (100) planes at representative 2θ value (Figure 1B). These two peaks are indicative of graphitic carbon materials with sp^2^ graphitic hybridization distortion [20,26,27]. It is also evident that SNPO-CPL with carbonization temperature 800 °C depicts a broader peak of (002) plane compared to 600 and 1000 °C. This variation in width of XRD peaks is attributed to the different crystallite size of the as-fabricated nanoplates [26]. Thus, SNPO-CPL-800 has improved crystal quality in terms of fine-tuned tiny crystal particles compared to its counterparts.

Raman spectra shows two distinct bands (Figure 1C) at around 1365 and 1580 cm^−1^, which correspond to the D and G bands, respectively [28]. The G (graphitic) band indicates a high level of graphitic carbon (sp^2^-hybridization), whereas the D (disorder and defects) band indicates sp^2^-hybridization caused by the doping of heteroatoms (S, N, P, and O) to the graphitic matrix. The value of the ID/IG ratio was observed to be 0.96, 1.11, and 1.21 for SNPO-CPL-600, SNPO-CPL-800, and SNPO-CPL-1000, respectively. The increased ratio of SNPO-CPL-1000 results from the high carbonization temperature which may lead to, (i) the removal of N, S-atoms, thus promoting structural defects in the graphitic matrix, (ii) narrowing of both the D and G bands, leading to expansion of the graphite structure in the crystalline area, and (iii) generation of numerous holes and defects because of pyrolysis of gases, which results in the formation of disordered carbons [29]. These findings suggested that the ID/IG proportion directs the number of imperfections, which is also in accordance with the reported literature. Additionally, under KOH activation, various types of pores are produced in the graphitic carbon, whereas crystal lattices are normally expanded through K ion intercalation [30]. We used a moderate weight ratio (KOH/carbon) of 1 for activation of the as-fabricated materials. The surface topography of the as-prepared SNPO-CPL was also determined via low temperature N_2_ absorption–desorption isotherm (Figure 1D). The BET surface areas of SNPO-CPL with carbonization temperature 600, 800, and 1000 °C were observed to be 140, 145, and 148 m^2^ g^−1^, respectively. The pore size was also calculated and is shown in Figure S4. The high surface area of SNPO-CPL-1000 could be due to smaller sized nanoplates.

X-ray photoelectron spectroscopy (XPS) of SNPO-CPL-800 was carried out not only to verify the doping but also to evaluate the chemical bonding states of the heteroatoms (S, N, P, and O) as depicted in survey spectrum (Figure S5A). Five characteristic peaks corresponding to the binding energies of C1s, O1s, N1s, S2P, and P2P were detected in high resolution XPS spectra as shown in Figure S5B–F. The deconvoluted high-resolution C1s peak illustrates C=C (288.6 eV), C-O (286.2 eV), C-N (285.1 eV), C-C (284.5 eV), and C1s 1 peak, positioned at 283.7 eV (Figure S5B) [31]. O1s peak is further split into three distinguished peaks at around 533.28, 531.89, and 530.19 eV corresponding to chemisorbed oxygen, ether or phenolic-type groups, and carboxylic group, respectively (Figure S5C) [32]. Moreover, Figure S5D reveals N1s spectrum with deconvoluted peaks at 401.2, 399.7, and 397.89 eV corresponding to pyridinic nitrogen, quaternary nitrogen, and pyrrolic/pyridinone nitrogen, respectively [33]. Similarly, deconvoluted 2p^3/2^ and 2P^1/2^ spectrum of S are depicted in Figure S5E. The peaks are fitted to three energy components centered at around 169.7, 168.4, and 163.3 eV indexing to S2P^1/2^, oxidized S, and S2P^3/2^, respectively [34]. P spectrum is deconvoluted into three component peaks centered at 132.89, 132.2, and 131.8 eV corresponded to P=O, P-O, and P-C bonding, respectively (Figure S5F) [20]. These results confirm that the O, N, S, and P heteroatoms were intrinsically doped into the graphitic carbon framework. The atomic percentage distribution obtained from XPS is shown in Table S1. The decrease in N and O content with increase in temperature can be due to de-nitrogenating and dehydrogenation crosslinking reactions, whereas the decrease in C content can be attributed to heat shrinking, contraction, or collapse of pores at high temperature, in accordance with the reported literature [35].

**Figure 2:**
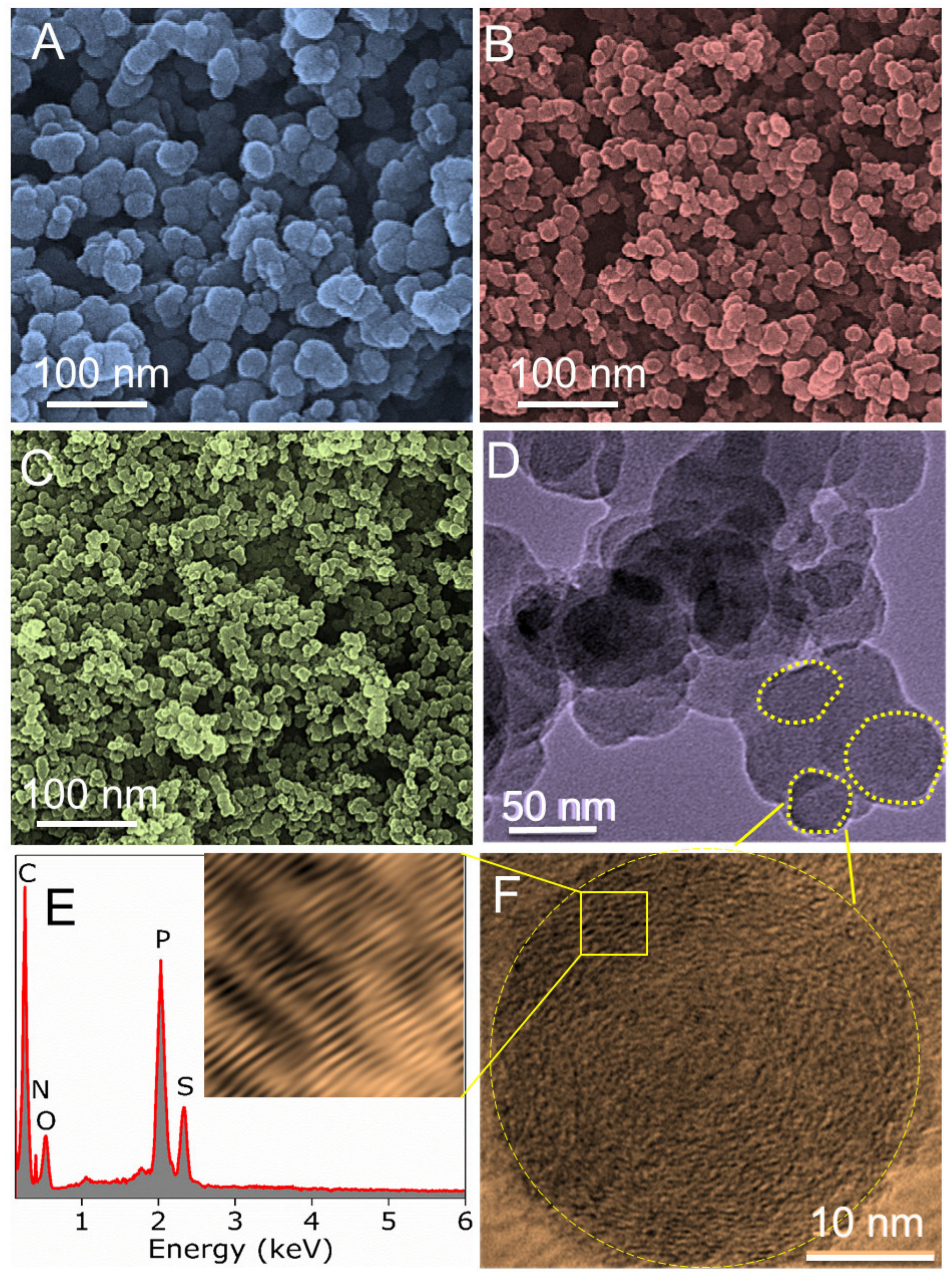
SEM and TEM images revealing homogeneity in SNPO-CPL nanoparticles. SEM images of (**A**) SNPO-CPL-600 (**B**) SNPO-CPL-800 and (**C**) SNPO-CPL-1000. (**D**) TEM images of SNPO-CPL-800 (**E**) EDS line spectrum (inset inverse-FFT) and (**F**) HRTEM of the SNPO-CPL-800.

Morphological analysis was performed through SEM and TEM characterizations. SEM observations revealed that by increasing the carbonization temperature from 600 to 1000 °C the size of the SNPO-CPL decreased directly (as shown in Figure 2A–C). For example, the average size of SNPO-CPL material was observed to be 30–35, 25–30, and 15–18 nm, respectively, at 600, 800, and 1000 °C carbonization temperature. This decrease in size can be attributed to de-nitrogenating, dehydrogenation, and heat shrinking, contraction, or collapse of pores at high temperature, as well as less thermal expansion due to constrained nanoplates [36].

Moreover, SEM–EDS peaks and mapping analysis further validated the presence of S, N, P, O, and C in SNPO-CPL (Figure 2E and Figure S6). The SNPO-CPL-800 was further probed through TEM analysis as shown in Figure 2D,F and inset panel Figure 2E. Analogous to SEM findings, the TEM investigations also revealed that after carbonization, the microspheres disintegrated into plate-like structures which were further composed of smaller irregular shaped fragments (Figure 2D). Moreover, the amorphous nature of plate-like graphitic carbon is also evident from the edges (Figure 2C), which is ascribed to heteroatom doping in the carbon lattice. An average lattice spacing of 0.36 nm was calculated by using FFT and inverse-FFT patterns and some structural defects were found, possibly due to the doping effect (inset panel Figure 2E). This increase in lattice spacing of C (covalent radius, 77 pm) is attributed to insertion of heteroatoms having a larger covalent radius; 102 and 107 pm for S and P, respectively. These findings confirm the effective insertion of heteroatoms N, S, P, and O along the lattice spacing of carbon, which is also in accordance with previous reports [27,37,38].

Figure 2A–C shows a magnified SEM top-view of SNPO-CPL-600, SNPO-CPL-800, and SNPO-CPL-1000, respectively, as well as histograms exhibiting the size distribution of the nanoplates (insets Figure 2A–C), wherein the size of the nanoplates decreases gradually upon increasing carbonization temperature. The SEM-EDS graph of SNPO-CPL-800 is shown in Figure 2E. A TEM image of SNPO-CPL-800 reveals plate-like structures (Figure 2D), with amorphous surface (Figure 2F) and increased lattice spacing value (inset panel Figure 2E).

### 3.3. Electrocatalytic Assessments of SNPO-CPL

**Figure 3:**
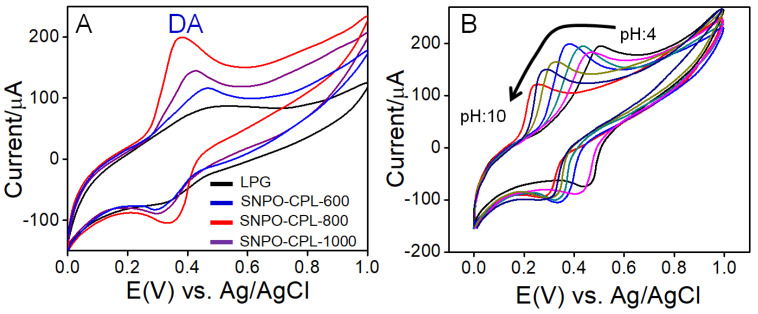
(**A**) CVs of SNPO-CPL-600 (blue line), SNPO-CPL-800 (red line), SNPO-CPL-1000 (purple line), and bare LPG (black line) toward monitoring of 5 µM of DA in N_2_ saturated 0.1 M PBS (pH 7) at scan rate 100 mVs^−1^. (**B**) CV of SNPO-CPL-800 over a pH range of 4 to 10 with scan rate of 100 mVs^−1^ in the presence of 5 µM of DA.

The electrocatalytic signaling efficiency of the SNPO-CPL-(600, 800, and 1000) was compared through cyclic voltammetry (CV) and electrochemical impedance measurements (Figure S7). As per results, SNPO-CPL-800 showed improved electrocatalytic efficacy compared to SNPO-CPL-600 and SNPO-CPL-1000 in terms of fast electron transportation, highly exposed catalytic active sites, and low resistance against charge transfer (see Supplementary Materials, Section S2.1). Similarly, electro-oxidation of DA was measured through CV at surfaces of all three electrodes SNPO-CPL-(600, 800, and 1000) and bare LPG in 0.1 M PBS (pH 7) at a scan rate of 100 mVs^−1^ in the absence and presence of 5 µM of DA (Figure 3A). A reversible oxidation–reduction peak was observed for DA at the interface of all four electrodes with varied peak separation values. Briefly, SNPO-CPL-800 exhibited higher peak current and smaller peak-to-peak separation (ΔEp) values (130.18 µA and 0.02 mV) compared to SNPO-CPL-1000 (73.04 µA and 0.10 mV), SNPO-CPL-600 (51.25 µA and 0.13 mV) and bare LPG (23.41 µA and 0.17mV). The high electrocatalytic efficacy of the SNPO-CPL-800 compared to SNPO-CPL-600 and SNPO-CPL-1000 could be attributed to, (i) the synergetic effect of a unique amalgam of hierarchically porous nanoplates with improved crystal quality and tuned crystal particles, as evidenced from XRD spectra (Figure 1B), (ii) the presence of more graphitic C and a significant number of surface flaws as per Raman results (Figure 1C), and (iii) the possibility that C-based matrices may transform to polarized surfaces when the most suitable or a high percentage of S, N, P, and O (as evidenced from Table S1) atoms are doped through the sp^2^-C chain with a large number of catalytic active sites, providing adequate housing and binding sites against target biomolecules and promoting electrocatalytic signaling.

The impact of pH value on the electrocatalytic performance of SNPO-CPL-800 toward DA oxidation was thoroughly investigated (Figure 3B). The electrochemical efficacy of SNPO-CPL-800 was measured over a pH range of 4 to 10 with a scan rate of 100 mVs^−1^ in the presence of 5 µM of DA. Results show that the anodic peak current (Figure S8A) rose with an increase in pH from 4 to 7 and then started decreasing from 7 to 10. At pH 7, the maximum anodic peak current was recorded. Thus, the electrocatalytic oxidation efficiency of SNPO-CPL-800 was observed in PBS with pH 7. Figure S8B represents a linear relation between pH and anodic peak potential (Ea) with R^2^ = 0.99. These findings demonstrate that the number of electrons involved in the redox process is equal to the number of protons [39].

The electro-oxidation mechanism of DA (5 µM) at the SNPO-CPL-800 electrode was further studied through CV measurements at increasing scan rate (20–200 mVs^−1^). Figure S9 indicates that the redox peak of DA increased with the increase in scan rate. Cathodic and anodic peaks showed a linear relationship with scan rates. These results further suggest that the electro-oxidation of DA on the surface of the proposed electrode is an absorption-control process. The concentration of DA, absorbed at the surface of the electrode could be calculated using Randles–Sevcik equation Equation (1) [34].

Ip = n − -2F2ℾvA/4RT(1)

**Scheme 2:**
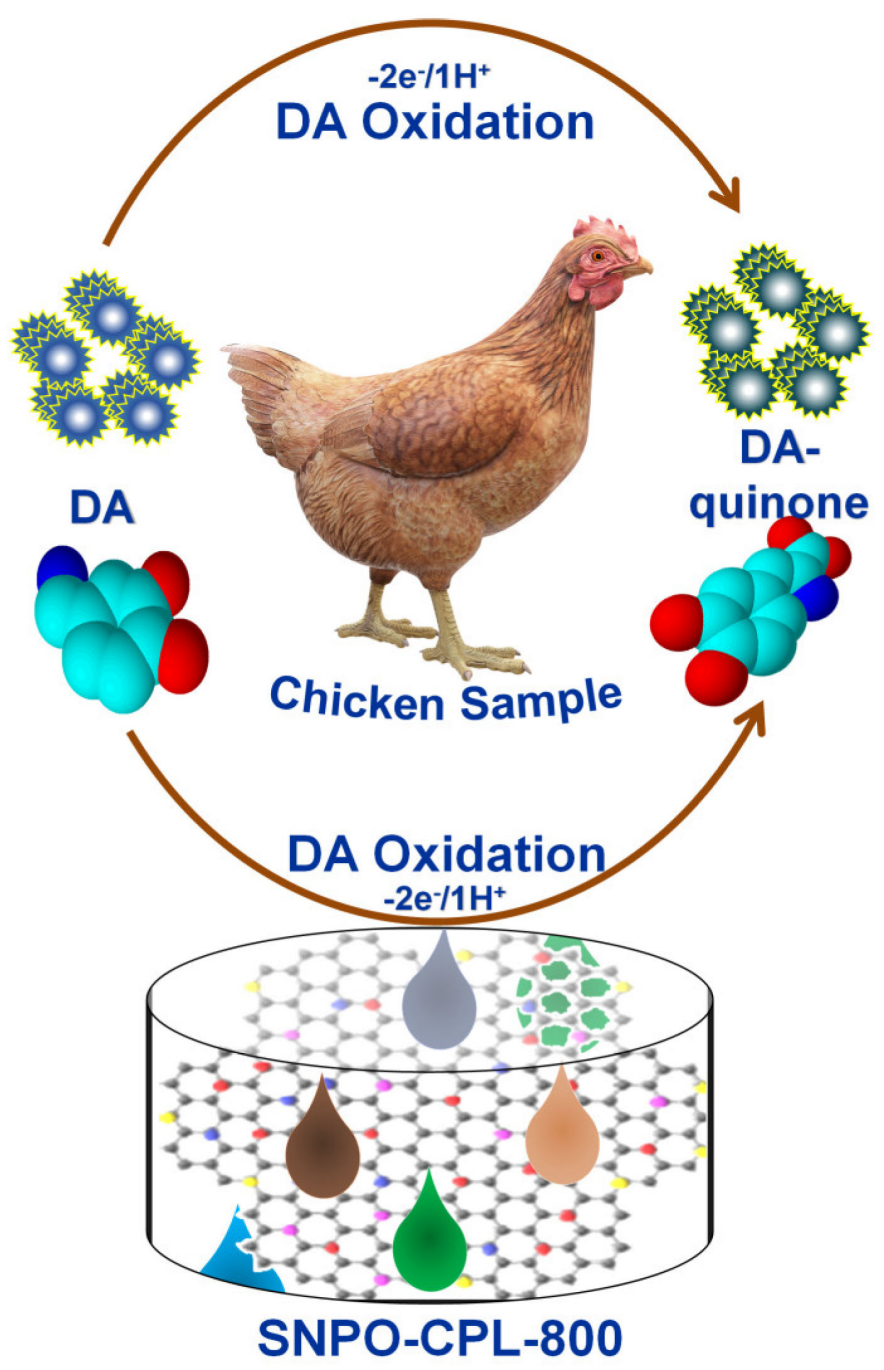
Schematic illustrations showing the oxidation of DA at the surface of SNPO-CPL-800.

Here n is the number of electrons involved during electro-oxidation, ν represents scan rate, F is the Faraday constant, A is the specific surface area of the LPG (49 × 10^−3^ cm^2^), R is the gas constant, T is the absolute temperature, and Γ shows the total concentration of DA at the surface of LPG. The value of Γ was calculated to be 6.48 × 10^−4^ mole/cm^2^, which was much higher than the N-doped mesoporous carbon sheets and sulfur-doped microporous carbon [34,39]. Ks denotes the electron transfer rate constant whereas α shows the charge transfer coefficient. To calculate these parameters, Laviron’s theory Equation (2) was applied [39]:

log ka/kc = log α/(1 − α) or (ka)/kc = α/(1 − α)(2)

where ka is the slope of calibration curve derived from Ea versus log v which is equal to 2.3 RT/(1 − α) nF. Similarly, kc is the slope of calibration curve derived from Ec versus log v which is equal to −2.3 RT/αnF. The calculated value of α was observed to be 0.391. By using Laviron’s theory, Equation (3) for the heterogeneous electron transfer rate constant (Ks) could also be calculated:

log ks = α log(1 − α) + (1 − α) log α − log RT/nFv − α(1 − α) nF ΔEp/2.3 RT(3)

where ΔEp is the difference between anodic and cathodic peak potential and n is the number of electrons evolved during the reaction. The value of ks was calculated to be 0.241 s^−1^. Using the same method, ks and α of bare LPG was also calculated and found to be 0.76 and 0.14 s^−1^, respectively. These results suggest that our developed SNPO-CPL-800 boosted the charge transfer more efficiently between electrode–electrolyte interfaces compared to bare LPG. The possible reaction mechanism for the oxidation of DA at the surface of the designed SNPO-CPL-800 electrode is shown in Scheme 2.

### 3.4. Sensing Efficacy of SNPO-CPL-800 toward DA Monitoring

**Figure 4:**
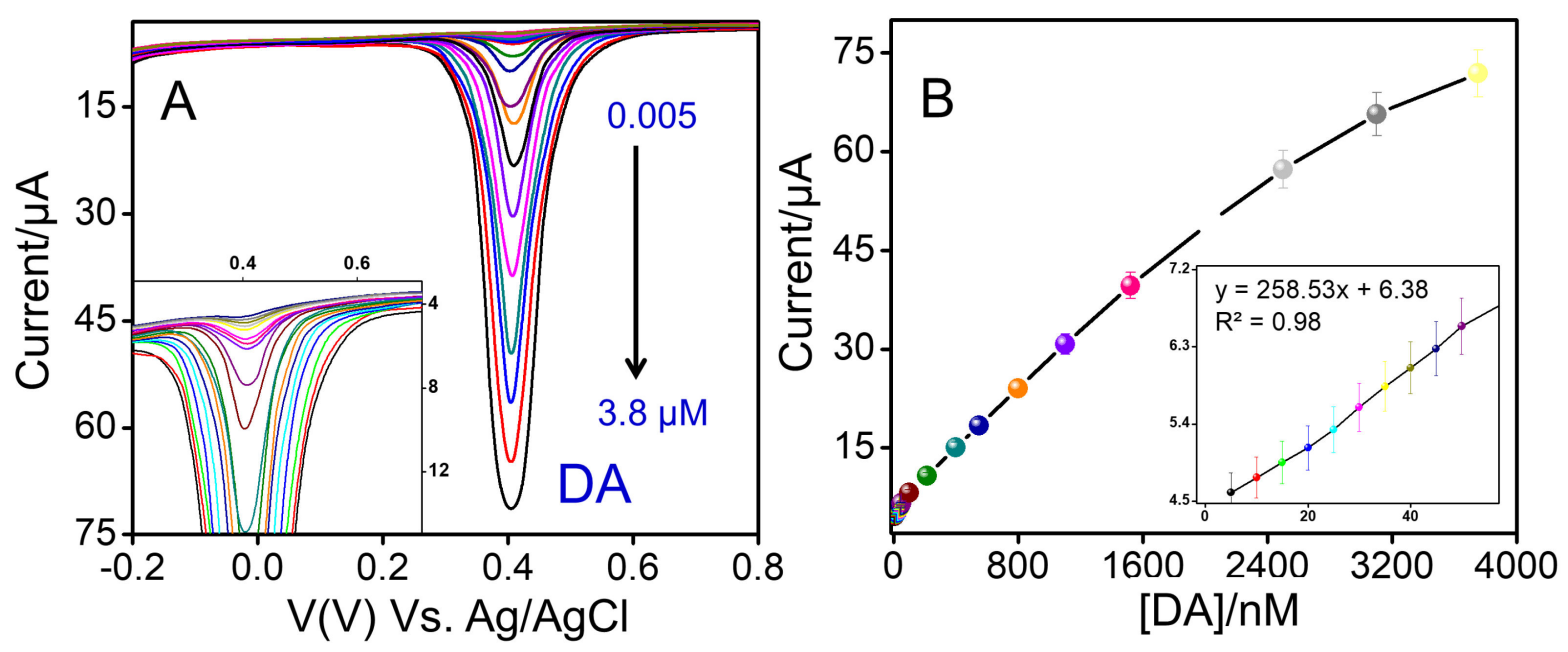
(**A**) DPVs of SNPO-CPL-800 toward increasing concentration of DA in 0.1 M PBS (pH 7) electrolyte. (**B**) Calibration curves (current versus concentration) of DA derived from their respective DPV curves.

To investigate the sensing efficacy of the SNPO-CPL-800 toward DA, we performed DPV measurements at a pulse height of 60 and pulse distance of 100 ms, at a constant scan rate of 100 mV/s (Figure 4) in 0.1 M PBS solution as an electrolyte with pH 7. Figure 4A shows the DPV spectra of SNPO-CPL-800 toward increasing concentration of DA. Results reveal that the peak current density rose linearly as the concentration increased. By plotting a calibration curve between concentration and current, a linear relationship was observed (Figure 4B) with regression equation I (µA) = 258.53 [DA] (nM) + 6.38 and correlation coefficient R^2^ = 0.98 (S/N =3). The detection limit was calculated and found to be 0.009 nM and linear range was 5–3850 nM.

The developed SNPO-CPL-800 electrode showed high reliability in terms of detection limit and sensitivity in comparison to previously reported N doped mesoporous carbon sheets and sulfur-doped microporous carbon, and other examples from the related literature (Table S2).

### 3.5. Selective Efficacy of SNPO-CPL-800 toward DA Monitoring

**Figure 5:**
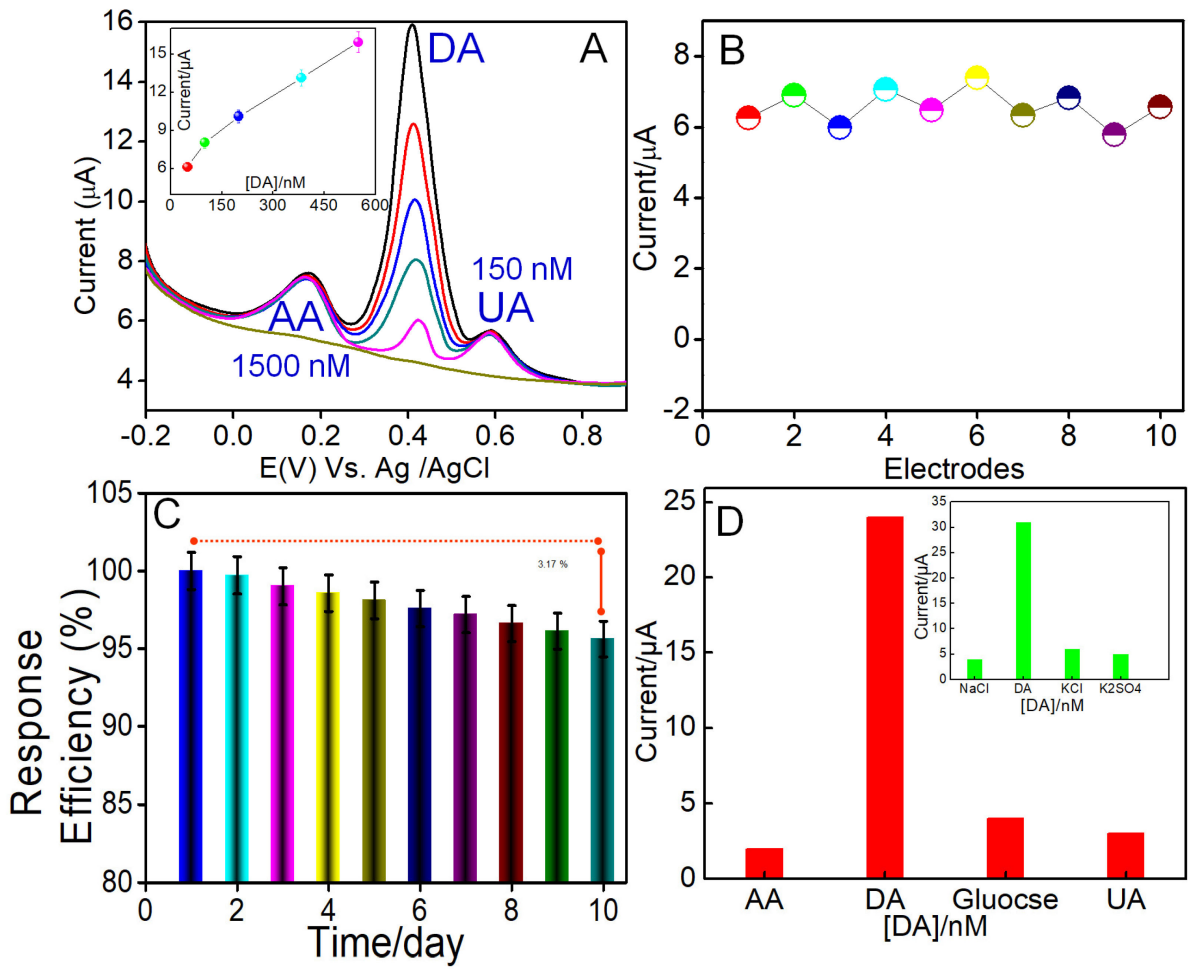
DPVs of SNPO-CPL-800 toward selective signaling of (**A**) DA with increasing concentration range 0.05 to 0.55 µM in the presence of constant concentration of AA (0.15 µM) and UA (1.5 µM). Inset of A shows the respective calibration curve derived from their DPV curves. (**B**) Reproducible current response of 10 different SNPO-CPL-800 electrodes toward 50 nM of DA, (**C**) Long-term stability of the SNPO-CPL-800 based electrode for continuous 10 days towards electrochemical oxidation of fixed concentration (50 nM) of DA through DPV, and (**D**) Amperometric selective current response of SNPO-CPL-800 based electrode toward chicken sample containing 0.8 µM of DA in the co-existence of 2 µM of AA, UA, and glucose interferences at an applied potential of 0.39 V. Inset of F shows amperometric selective current response of SNPO-CPL-800 based electrode toward chicken sample containing 1.1 µM of DA in the co-existence of 5 µM of inorganic interferences such as NaCl, KCL, and K_2_SO_4_ at an applied potential of 0.39 V.

The portable non-enzymatic electrochemical sensor has revolutionized modern society in terms of early medicinal diagnosis, public healthcare, and food safety evaluation. However, designing non-enzymatic electrochemical sensors for real-time monitoring of DA is quite intriguing due to the co-existence of other bioactive interfering agents such as AA and UA, which can also generate electrical signals along with the DA. For real-time monitoring, selectivity is one of the major pillars in designing non-enzymatic electrochemical sensors for DA. Thus, DPV measurements were carried out to evaluate the selectivity response of the designed SNPO-CPL-800 electrode toward DA monitoring in the co-existence of UA and AA. DPV analysis was carried out in 0.1 M PBS (pH 7) electrolyte by increasing the concentration of DA while keeping the concentrations of the other two analytes constant (Figure 5). Briefly, Figure 5A depicts the oxidation potential of DA at 0.39 V (Ag–AgCl), which is directly correlated with the rising concentration of DA, even in the presence of fixed concentrations of AA (1500 nM) and UA (150 nM). The calibration curve (inset Figure 5B) shows that SNPO-CPL-800 displayed linear behavior over a concentration range of 50–550 nM.

Thus, the designed SNPO-CPL-800 electrode shown remarkable selectivity efficiency, as evident from Figure 5A, with three components. AA and DA have a distinct peak potential difference (E = 0.02 V) as do DA and UA (E = 0.021 V), respectively. Selectivity efficacy of the designed SNPO-CPL-800 electrode could be ascribed to: (i) the presence of a large number of active sites thus resulting in the provision of appropriate lodging and binding sites and (ii) the possibility that disclosing a higher proportion of active sites along with firmly interacted graphitic C, and along with S, N, P, and O, reinforces the analyte species at their particular oxidation potential. These findings suggest that the designed electrode has strong potential in terms of practical usability.

### 3.6. Reproducible Efficiency of the Designed SNPO-CPL-800 Electrode

To evaluate reliability of the designed SNPO-CPL-800 electrode various factors including reproducibility, reusability, and long-term stability were also monitored through DPV by following the reported standard procedure [40]. Reproducibility of 10 electrodes was assessed by measuring electro-oxidation of DA. The results showed RSD fell within the range of 1.21–2.20% against 50 nM of DA (Figure 5B). Further, the stability of the SNPO-CPL-800 electrode was monitored consistently for 10 days towards DA (50 nM) electro-oxidation. Even after the 10th day, the results showed a negligible (5.2%) decrease in oxidation peak current response (Figure 5C).

### 3.7. DA Monitoring in Chicken Samples

Apart from brain chemistry, monitoring the DA from chicken samples is of great demand not only to assess the drug’s efficacy but also the quality of meat and chicken. DA in powder form is supplemented to the animal feed illegally as an adrenergic neural stimulant to stimulate and build muscle rather than fat. Therefore, we selected DA spiked chicken samples for real-time evaluation of our developed electrode. Here, we used our designed SNPO-CPL-800 electrode for the first time for the real-time monitoring of DA from commercially available chicken through amperometric measurements (Figure 5D) by following our already reported strategy [41]. Amperometric measurements clearly showed the selective peak current values of ~23.1 and ~30.13 µA towards 0.8 and 1.1 µM of DA even in the co-existence of inorganic (inset Figure 5D) and organic (Figure 5D) reagents. These results support the electrode’s remarkable reliability in terms of practical application.

## 4. Conclusions

We have reported a facile, green synthesis of a novel, highly selective and sensitive electrochemical sensor for detection of DA using a metal-free electrode based on heteroatom-doped carbon nanoplates (SNPO-CPL). We fabricated SNPO-CPL electrodes by simple carbonization of heteroatoms (S, N, P, O) containing poly(cyclcotriphosphazene-co-2,5-dioxy-1,4-dithiane) (PCD) polymer at three different temperatures (600, 800, and 1000 °C). The SNPO-CPL material carbonized at 800 °C (SNPO-CPL-800) showed better electrocatalytic efficiency in terms of electron transportation and better conductivity compared to SNPO-CPL-600 and SNPO-CPL-1000, mainly because of the better crystal quality and well-tuned crystal particles, high surface area, optimized doping of S, N, P, and O heteroatoms through the sp2-carbon chain, and large surface defects. The developed SNPO-CPL-800 electrode was successfully employed for the sensitive screening of DA even in the presence of co-existing interferences. To evaluate the reliability, the developed SNPO-CPL-800 electrode was also used to monitor DA from commercially available chicken samples.

## Data Availability

Not applicable.

## Supplementary Materials

The following supporting information can be downloaded at: https://www.mdpi.com/article/10.3390/bios12121106/s1, Figure S1. Digital image of LPG system prepared for electrochemical measurements. Figure S2. XRD analysis of PCD polymer. Figure S3. SEM image of PCD polymer revealing sphere like morphology. Figure S4. Pore size distribution of all three electrodes. Figure S5. (A) Survey spectrum of SNPO-CPL-800. Deconvoluted high resolution XPS spectrum of C (B), O (C), N (D), S (E) and P (F). Figure S6. (A) Mapping analysis of SNPO-CPL-800 with homogeneous distribution (A) of S (B), N (C), P (D), O (E) and C (F). Figure S7 shows CV (A) and typical-Nyquist impedance spectra (B) for SNPO-CPL-600, SNPO-CPL-800, and SNPO-CPL-1000 in 5 mM [Fe(CN)6] in 0.1 M PBS (pH 7). Table S1. Atomic percentages of the as synthesized electrocatalysts from XPS analysis. Figure S8. (A) Peak potential versus pH and (B) plot of anodic current versus pH derived from Figure 3B. Figure S9. (A) CV of SNPO-CPL-800 with varying scan rate from 20–100 mV/s. (B) Peak current vs. scan rate derived from Figure S9A. Table S2. Sensitivity and limit of detection comparison of different carbon-based electrodes for DA. References [42,43,44,45] are cited in Supplementary Materials.

## Abbreviations

Ascorbic acid (AA) Dopamine (DA), 1,4-dithiane-2,5-diol (DD), Fourier Transform Infrared (FTIR), Field emission scanning electron microscopy (FE-SEM), Energy dispersive X-ray spectroscopy (EDX), Lead pencil graphite (LPG), Powder X-ray diffraction (PXRD), Poly(cyclcotriphosphazene-co-2,5-dioxy-1,4-dithiane) (PCD), Limit of detection (LOD), S, N, P, and O doped carbon nanoplatelets (SNPO-CPL), Hexachlorocyclotriphosphazene (HCCP), Poly(cyclcotriphosphazene-co-2,5-dioxy-1,4-dithiane) (PCD), Tetrahydrofuran (THF), Potassium hydroxide (KOH), Triethylamine (TEA), Phosphate buffer soln. (PBS), Uric acid (UA), X-ray photoelectron spectroscopy (XPS).
