# Supplementary material for: Cyclophosphazene Intrinsically Derived Heteroatom (S, N, P, O)-Doped Carbon Nanoplates for Ultrasensitive Monitoring of Dopamine from Chicken Samples
Source: Biosensors (Basel). 2022 Dec 1;12(12):1106. doi: 10.3390/bios12121106 (PMC9776138; doi:10.3390/bios12121106)
Supplement: Supplementary file 1 [file biosensors-12-01106-s001.zip › biosensors-1973839-supplementary.pdf]

biosensors

MDPI

*Article*

# Cyclophosphazene Intrinsically Derived Heteroatom (S, N, P, O)-Doped Carbon Nanoplates for Ultrasensitive Monitoring of Dopamine from Chicken Samples

**Yasir Abbas [1,†], Naeem Akhtar [2,†], Sania Ghaffar [3,†], Ahlam I. Al-Sulami [4], Muhammad Asad [5], Muhammad Ehsan Mazhar [6], Farhan Zafar [7], Akhtar Hayat [8,*] and Zhanpeng Wu [1,*]**

1 Laboratory of Carbon Fiber and Functional Polymers (Beijing University of Chemical Technology), Ministry of Education, Beijing 100029, China
2 Institute of Chemical Sciences, Bahauddin Zakariya University (BZU), Multan 60800, Pakistan
3 Nishtar Medical University, Multan 60800, Pakistan
4 University of Jeddah, College of Science, Department of Chemistry, Jeddah 21589, Saudi Arabia
5 Department of Materials Science and Engineering, University of Science and Technology of China No. 96 Jinzhai Road, Hefei 230026, China
6 Department of Physics, Bahauddin Zakariya University (BZU), Multan 60800, Pakistan
7 Department of Chemistry, COMSATS University Islamabad, Lahore Campus, Lahore 54000, Pakistan
8 Interdisciplinary Research Centre in Biomedical Materials (IRCBM), COMSATS University Islamabad, Lahore 54000, Pakistan
* Correspondence: akhtarhayat@cuilahore.edu.pk (A.K.); wuzp@mail.buct.edu.cn (Z.W.)
‡ These authors have contributed equally to this work.

**Supplementary Materials:**

## 1. Experimental Section

### 1.1 Characterization Analyses

Polymerization was confirmed via Fourier Transform Infrared (FTIR) by using a Bruker Vertex 70 FTIR spectrometer. The morphology of as prepared samples was investigated using Field emission scanning electron microscopy (FE-SEM, S-4800, JEM-6701F). Energy dispersive X-ray spectroscopy (EDX) coupled with FE-SEM was used to determine the elemental percentages. Microstructures of graphitic materials were carried out on FEI Tecnai T-20 TEM. It provides information about the morphology; dimensions and crystallinity as well as defects and crystal orientations. To analyze the crystal structures, a diffractometer (XRD-6000) was used to characterized for powder X-ray diffrac-tion (PXRD) at voltage of 40 kV using monochromated Cu K$\alpha$ radiation ($\lambda$ = 1.54 Å, 40 kV, 30 mA) at scan rate of 10° min$^{-1}$. Raman spectroscopy is a simple but versatile technique used to identify rotational, vibrational, and other lower energy modes in the materials. Using Raman (Renishaw invia microscope), we studied ratio of in-plane vibrations of the sp$^2$ carbon (G-band) and disorder-induced mode (D-band) at excitation wavelength of 633 nm. The $N_2$ adsorption-desorption were measured via a Quantachrome Autosorb-1C-VP. Prior to $N_2$ adsorption-desorption, all the sample were subjected at 200 ˚C under vacuum for 5 hours. Specific surface area was determined via Brunauer−Emme −Teller method. X-rays photoelectron spectroscopy (XPS) is surface sensitive spectroscopic techniques used to measure the chemical states surface compositions, and electronic states. XPS was performed (using K-Alpha, Thermo Fisher Scientific, USA) coupled to an ultrahigh vacuum system equipped with a monochromatic Al K$\alpha$ (1486.6 eV).

2. Results and discussion

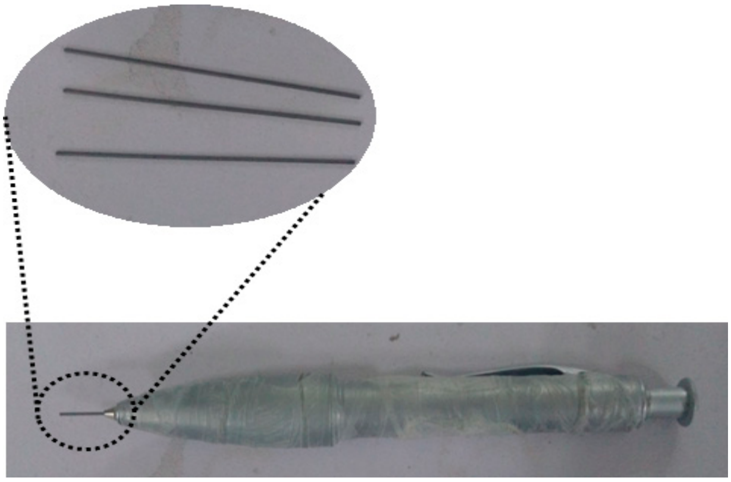

Figure S1. Digital image of LPG system prepared for electrochemical measurements.

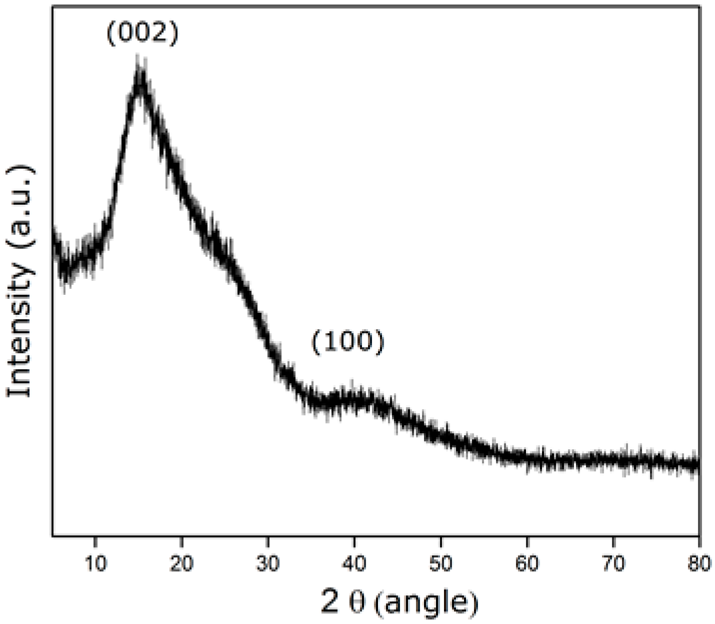


Figure S2. XRD analysis of PCD polymer.

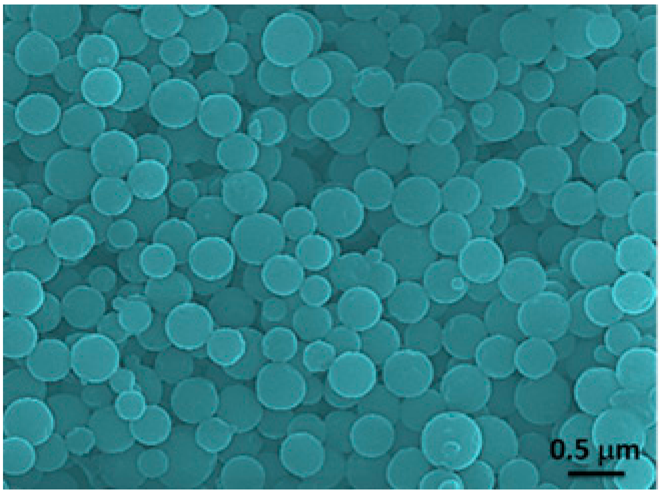


Figure S3. SEM image of PCD polymer revealing sphere like morphology.

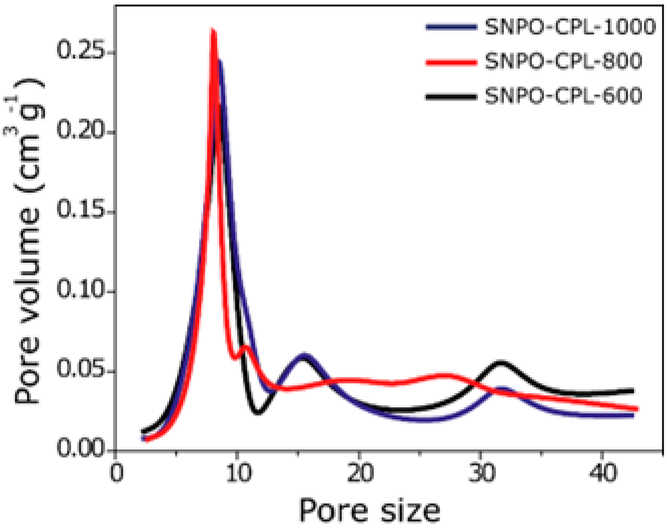


Figure S4. Pore size distribution of all three electrodes.

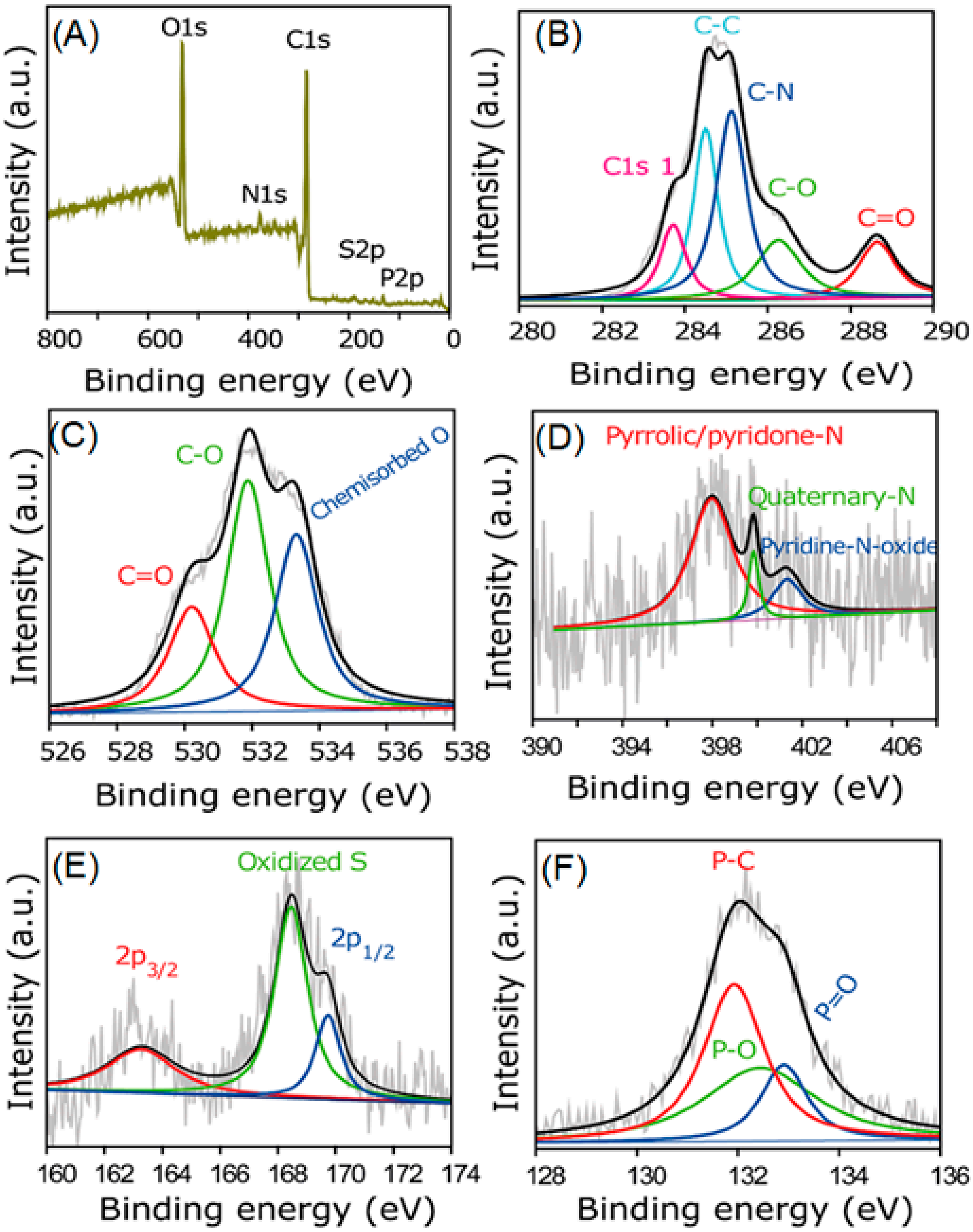


Figure S5. (A) Survey spectrum of SNPO-CPL-800. Deconvoluted high resolution XPS spectrum of C (B), O (C), N (D), S (E) and P (F).

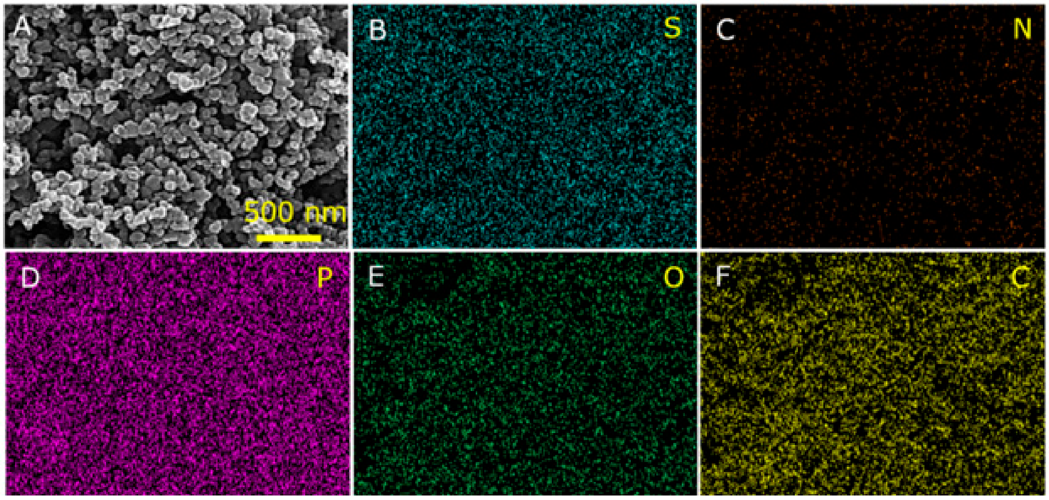


Figure S6. (A) Mapping analysis of SNPO-CPL-800 with homogeneous distribution (A) of S (B), N (C), P (D), O (E) and C (F).

2.1 The Electrochemical Activity of SNPO-CPL-(600, 800 and 1000)

The electrochemical activity of SNPO-CPL-(600, 800 and 1000) modified LPG was assessed through cyclic voltammogram (CV) measurements in 0.1 M PBS electrolyte containing 5 mM [Fe(CN)$_6$] with in applied potential rang of 0.0 to 0.75 V. A pair of redox peak with varying peak current and potential appeared at the surface of all four electrodes (Figure S7A). However, SNPO-CPL-800 shows high peak current and small peak separation difference compared to SNP-CPL-600 and SNP-CPL-1000. This high sensing efficacy of SNP-CPL-800 could be attributed to efficient charge density at the delocalized surface because of maximum percentage doping of S, N, P, O, and graphitic C.

Additionally, charge transfer capability of the SNPO-CPL-(600, 800 and 1000) were also calculated by impedance spectroscopy (EIS). Figure S7B shows the Nyquist plot of impedance spectrum with two portions; semicircle at high frequency refers the electron transfer resistance (Res) and line part at low frequencies reflects the diffusion process velocity. Results indicate that SNP-CPL-800 have the smallest semicircle diameter compared to others, which indicate high electron transport efficiency of the developed electrode.

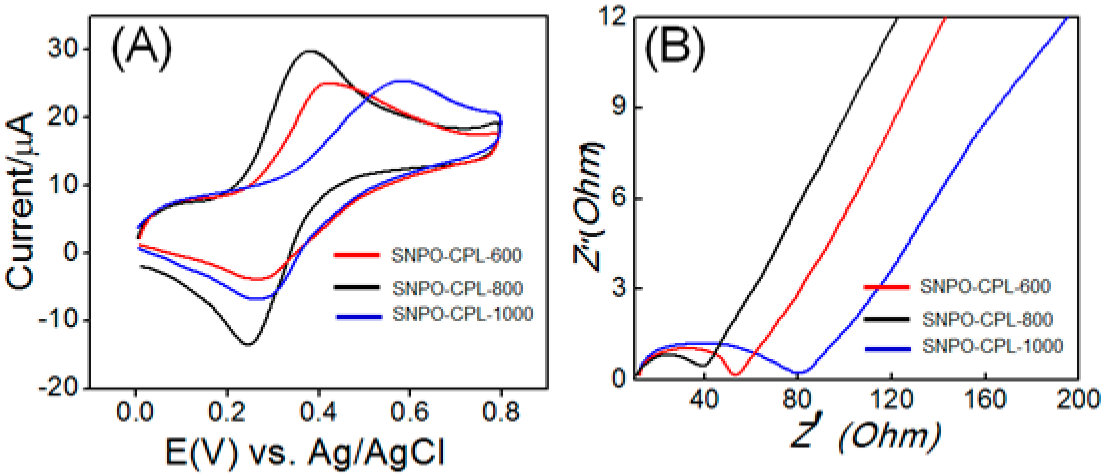


Figure S7. Shows CV (A) and typical -Nyquist impedance spectra (B) for SNPO-CPL-600, SNPO-CPL-800, and SNPO-CPL-1000 in 5 mM [Fe(CN)6] in 0.1 M PBS (pH:7).

Table S1. Atomic percentages of the as synthesized electrocatalysts from XPS analysis.

| Catalyst | Atomic % (S) | Atomic % (N) | Atomic % (P) | Atomic % (O) | Atomic % (C) |
|---|---|---|---|---|---|
| **SNPO-CPL-600** | 0.63 | 1.99 | 1.7 | 21.89 | 73.75 |
| **SNPO-CPL-800** | 0.87 | 2.02 | 1.87 | 22.3 | 72.98 |
| **SNPO-CPL-1000** | 0.00 | 0.00 | 0.88 | 14.24 | 71.84 |

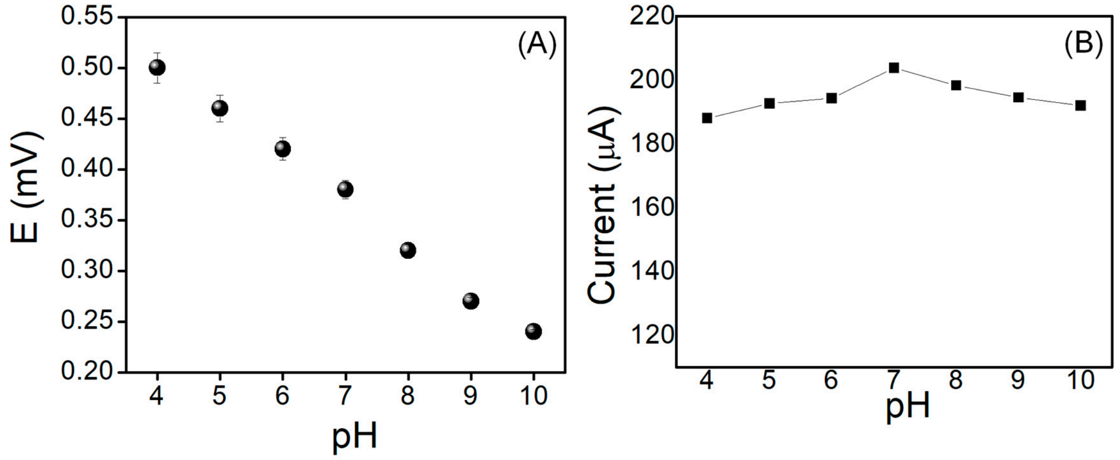


Figure S8. (A) Peak potential versus pH and (B) plot of anodic current versus pH derived from Figure 3B.

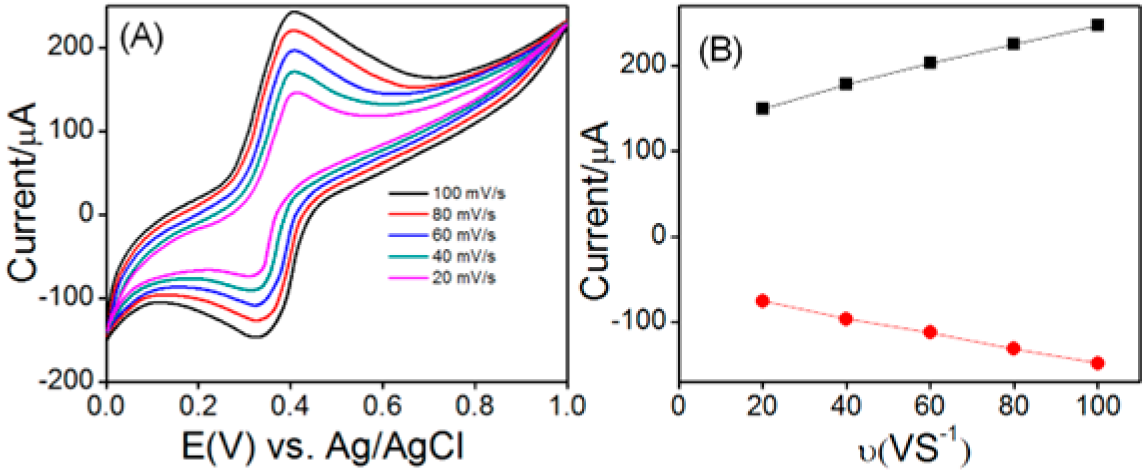

Figure S9. (A) CV of SNPO-CPL-800 with varying scan rate from 20- 100 mV/s. (B) Peak current vs. scan rate derived from figure S9-A.

Table S2. Sensitivity and limit of detection comparison of different carbon-based electrodes for DA.

| Electrocatalysts | Sensitivity (µA/mM) | Limit of detection (nM) | References |
|---|---|---|---|
| **Carbon nano-rods** | 5 | 60 | [1] |
| **Graphene oxide nanorib-bons/GCE** | 2.39 | 80 | [2] |
| **S doped carbon** | 4.1 | 3 | [3] |
| **N-doped gra-phene** | 9.87 | 1 | [4] |
| **N Gra-phene/GCE** | 0.113 | 250 | [5] |
| **N doped gra-phine** | 2.22 | 630 | [6] |
| **N doped gra-phine** | __ | 930 | [7] |
| **N, S doped car-bon** | __ | 0.02 | [8] |
| **P doped gra-phene** | __ | 0.36 | [9] |
| **N, P doped car-bon** | 7.94 | 600 | [10] |
| **SNPO-CPL-800** | 0.38 | 0.009 | This work |

Limit of detection calculated by using equation S1.

LOD = F x SD/*b*……………………………………….. (S1)

Where

F: Factor of 3.3, SD: Standard deviation of the blank, standard deviation of the ordinate intercept, or residual standard deviation of the linear regression, b: Slope of the regression line

## References


1. Demuru, S., et al., *Scalable Nanostructured Carbon Electrode Arrays for Enhanced Dopamine Detection.* ACS sensors, 2018. **3**(4): p. 799-805.
2. Sun, C.-L., et al., *Microwave-assisted synthesis of a core–shell MWCNT/GONR heterostructure for the electrochemical detection of ascorbic acid, dopamine, and uric acid.* Acs Nano, 2011. **5**(10): p. 7788-7795.
3. Emran, M.Y., et al., *One-step selective screening of bioactive molecules in living cells using sulfur-doped microporous carbon.* Biosensors and Bioelectronics, 2018. **109**: p. 237-245.
4. Feng, X., et al., *Three-dimensional nitrogen-doped graphene as an ultrasensitive electrochemical sensor for the detection of dopamine.* Nanoscale, 2015. **7**(6): p. 2427-2432.
5. Sheng, Z.-H., et al., *Electrochemical sensor based on nitrogen doped graphene: Simultaneous determination of ascorbic acid, dopamine and uric acid.* Biosensors and Bioelectronics, 2012. **34**(1): p. 125-131.
6. Wiench, P., et al., *Beneficial impact of oxygen on the electrochemical performance of dopamine sensors based on N-doped reduced graphene oxides.* Sensors and Actuators B: Chemical, 2018. **257**: p. 143-153.
7. Li, S.-M., et al., *Controllable synthesis of nitrogen-doped graphene and its effect on the simultaneous electrochemical determination of ascorbic acid, dopamine, and uric acid.* Carbon, 2013. **59**: p. 418-429.
8. Gao, W., et al., *One-Step Pyrolytic Synthesis of Nitrogen and Sulfur Dual-Doped Porous Carbon with High Catalytic Activity and Good Accessibility to Small Biomolecules.* ACS Applied Materials & Interfaces, 2014. **6**(21): p. 19109-19117.
9. Zhang, X., et al., *Phosphorus-doped graphene-based electrochemical sensor for sensitive detection of acetaminophen.* Analytica Chimica Acta, 2018. **1036**: p. 26-32.
10. Zhao, L., et al., *Electropolymerization fabrication of three-dimensional N, P-co-doped carbon network as a flexible electrochemical dopamine sensor.* Sensors and Actuators B: Chemical, 2017. **253**: p. 1113-1119.
